# Effects of Methylene Tetrahydro Folate Reductase Gene Polymorphisms on Methotrexate Toxicity in Egyptian Pediatric Acute Lymphocytic Leukaemia Patients

**DOI:** 10.22037/ijpr.2020.1101296

**Published:** 2020

**Authors:** Gomaa Mostafa-Hedeab, Yasser Elborai, Gamal Thabet Ali Ebid

**Affiliations:** a *Department of Pharmacology, Faculty of Medicine, Beni-Suef University, Egypt. *; b *Deparment of Pharmacology, Faculty of Medicine, Jouf University, Saudia Arabia. *; c *Department of Pediatric Oncology, National Cancer Institute, Cairo University, Egypt. *; d *Department of Clinical Pathology, National Cancer Institute, Cairo University, Egypt.*

**Keywords:** Methotrexate, Egyptian, *MTHFR*, Polymorphisms.

## Abstract

This study was designed to evaluate the effect of Methylenetetrahydrofolate reductase (*MTHFR)* gene polymorphisms on MTX toxicity in pediatric Egyptian ALL patients. Ninety-Four of Pediatric ALL patients aged 3–13 years (7.6 ± 3.6) on oral maintenance dose of 50 mg/m^2^ weekly of MTX. MTHFR c.677C>T (rs1801133) and c.1298A>C (rs1801131) genotyping were performed using polymerase chain reaction restriction fragment length polymorphism (PCR-RFLP). The allele frequencies of *c.677C>T *were 42.6%, 46.8%, and 10.6% for* CC, CT*, and *TT *respectively*,* while c.1298A>C alleles frequencies were 62.7%, 24.5%, and 12.8% for *AA, AC*, and *CC *respectively. None of the investigated polymorphism (*C677T* or *A1298C* alleles) was associated with either overall or site specific MTX toxicity regarding anemia (*p = *0.99) (*p = *0.4), platelets (*p = *0.4) (*p = *0.4), hepatotoxicity (*p = *0.4) (*p = *0.7), respectively. The results indicated that between *c.677C>T* genotypes, *CC/CT *and* TT *were associated with hematopoietic toxicities 60.7% and 60% (*p = *0.2); platelet toxicities 76.2% and 80% (*p = *1) and, hepatotoxicities 40.5% and 60% (*p = *0.3), respectiv*ely*. In the* c.1298A>C* genotypes, *CC/AC* and AA presented hematopoietic toxicities 68.6% and 55.9% (*p = *0.2), platelet toxicities 82.9% 72.9% (*p = *0.3) and, hepatotoxicity 37.1% and 45.8% (*p = *0.5), respectively. No significant associations were detected between *MTHFR* *c.677C>T* or *c.1298A>C* polymorphisms and either overall or site specific MTX toxicity.

## Introduction

Acute Lymphocytic Leukaemia (ALL) is the most common malignancy in childhood; it represents 30% of all pediatric malignancies. In the last 20 years, event-free survival was around 80% in the developed countries (1). Methotrexate is an important component of ALL treating protocols in childhood. Although MTX has an important successful role, toxicity may cause reduction or cessation of its dose. Therefore, predicting the adverse effect of MTX is crucial in treating ALL pediatric patients (2). 

Methotrexate is a pro-drug which requires intracellular polyglutamation for maximum cytotoxic effects. Methylenetetrahydrofolate reductase (MTHFR) catalyzes the irreversible conversion of dihydrofolate (DHF) to tetrahydrofolate (THF), an active form of folate needed for the *de novo* synthesis of the nucleoside thymidine is required for DNA synthesis and also essential for purine and pyrimidine base biosynthesis (3). Methotrexate, therefore, inhibits the synthesis of DNA, RNA, thymidylates, and proteins (4). 

MTX also affects other important enzymes, such as MTHFR; MTHFR is a key enzyme for intracellular folate homeostasis and metabolism (5). MTHFR converts the irreversible conversion of 5,10-methylenetetrahydrofolate (5,10-CH_2_-THF) to 5-methyltetrahydrofolate (5-CH_3_-THF), which is the predominant circulating form of folate and serves as the carbon donor for the remethylation of homocysteine to methionine (6, 7). Alterations in reduced folate pools, as a consequence of changes in MTHFR activity, may have a significant effect on the responsiveness of malignant and non-malignant cells to MTX. Accordingly, it has been proposed that impaired conversion of 5,10-CH_2_-THF to 5-CH_3_-THF and the subsequent modification in the intracellular folate pool could increase the toxic effect of MTX (8).

The MTHFR gene is localized on chromosome 1p36.3, *C677T*, and A1298C polymorphisms are two important single nucleotide polymorphisms (SNPs) of MTHFR (9). The* c.677C>T* allele encodes proteins with decreased enzymatic activity, in comparison with the normal allele 677C. People with the 677CT (heterozygous) and 677TT (homozygous) genotypes exhibit 60% and 30% of the activity of the normal homozygous 677CC genotype, respectively (10, 11). In the MTHFR A1298C polymorphism, the 1298C allele is responsible for a milder decrease in MTHFR activity with respect to the normal allele 1298A. The 1298CC homozygous individuals have 60% of the normal activity of 1298AA homozygous individuals (12). The effect of MTHFR gene polymorphisms on MTX induced toxicity is still receiving clinical studies and explanations, with conflicting results regarding polymorphism regulating intracellular MTX metabolic pathway and toxicity of MTX in pediatric ALL (13-16). 

Since *MTHFR* polymorphisms may affect sensitivity to MTX and their frequency may differ by ethnicity, we evaluated these polymorphisms in a group of Egyptian pediatric ALL patients on methotrexate-based maintenance treatment to determine the respective genotype frequencies and their impact on MTX toxicity.

## Experimental

*Study Population*

The present study included 94 ALL patients aged 3–13 years (mean 7.6 ± 3.6 years); 58 boys (61.7%) and 36 girls (38.3%). They presented to the pediatric oncology department, National Cancer Institute (NCI), Cairo University, Egypt. Diagnosis of ALL was performed according to clinical, morphological, cytochemical, and immunophenotyping examination. The patients included in the study received oral MTX as maintenance therapy at a dosage of 50 mg/m^2 ^weekly. The patients were followed up for at least six weeks. Institute review board (IRB) approval was obtained and data were stored in a password-protected database. Their immunophenotypes were pre-B-lineage in 83 (88.3%) and T-lineage in 11 (11.7%). According to the protocol risk stratification criteria, 57 patients (60.6%) were classified as low risk, whereas 37 (39.4%) were classified as standard risk.

*Methods*

The patients were followed up for 6 weeks after initiation of maintenance dose of MTX 50 mg/m^2^ weekly for bone marrow suppression and hepatic toxicity. At the end of 6^th^ week, White blood cells (WBCs) count with differential was collected to assess bone marrow suppression. To assess hepatic toxicity, the following were collected: alanine transaminase (ALT), aspartate aminotransferase (AST), total bilirubin (TB), and lactate dehydrogenase (LDH). Common terminology criteria for adverse events (CTCAE v. 4.03) were used to hemoglobin (Hgb) < 10.0 g/dL. Toxicity of MTX on platelets was considered if platelet count was <50,000 (10^3^/mm^3^). Hepatotoxic effect of MTX was defined as maximum ALT > 60 (2x ULN), maximum AST > 80 (2x ULN), and maximum TB > 2 mg/dL.

*MTHFR Genotyping*

A five-milliliter blood sample was withdrawn in an EDTA-coated tube from each patient, stored at -25 °C for genotyping. DNA was isolated from peripheral blood at diagnosis (16, 17). The *MTHFR C677T *and* A1298C* polymorphisms were identified using the method described by (18). After initial denaturation for 10 min at 95 °C, the PCR was performed for 35 cycles of 45 sec at 95 °C, 45 sec at 59 °C, and 1 min at 72 °C. The last elongation step was extended to 7 min (18). 

The amplified fragments targeted the sites of polymorphisms: the 198-bp fragment for *MTHFR C677T* containing the *C→T* bp substitution at nucleotide *677* that creates a HinfI restriction site and the 163-bp fragment for *MTHFR* *A1298C* contains the *A→C* substitution at nucleotide *1298* that abolishes a MboII restriction site. Therefore, HinfI and MboII (New England BioLabs, Beverly, MA) were used to detect the *C677T* and *A1298C* polymorphisms, respectively. The digestion products were visualized with ethidium bromide after electrophoresis on 3% agarose gel at 100 volts for 30 min for the *C677T* polymorphism and 4% agarose gel for the *A1298C* polymorphism. 

The *MTHFR 677CC* wild type homozygous was identified by the presence of only a 198 bp fragment. The *677CT* heterozygous was identified by 198, 175, and 23 bp fragments, and *the 677TT* homozygous was identified by 175 and 23 bp fragments. The *1298AA* wild type homozygous produces five fragments of 56, 31, 30, 28, and 18 bp. The *1298AC* heterozygous produces six fragments of 84, 56, 31, 30, 28, and 18 bp, and the *1298CC* homozygous variant produces four fragments of 84, 31, 30, and 18 bp.

*Statistical analysis*

Data were collected, tableted. Correlation of *MTHFR* alleles with clinical characteristics (WBC, cytogenetic risk, therapy-related toxicity) was also performed using the $\chi^{2}$ or Fisher’s exact test. All statistical calculations were done using Microsoft Excel 2010 (Microsoft Corporation, NY, USA) and SPSS (Statistical Package for the Social Science; SPSS Inc., Chicago, IL, USA) version 17 for Microsoft Windows. The level of significance was considered if *p *< 0.05.

## Results

The distribution of *C677T* and *A1298C MTHFR* gene frequencies among the study population are shown in Table 1.

*MTHFR C677T polymorphism and toxicity in pediatric ALL*

There was no significant difference in MTX toxicity between patients with anemia and those who had normal hemoglobin levels with different alleles (*p = *0.99). Within the patients of different alleles, there was no significant difference between the patients who had low platelets and those who had normal platelets (*p = *0.4). There was no significant difference between patients with different alleles related to hepatotoxicity (*p = *0.4) (Table 2).

At *MTHFR C677T* genotype, from the 94 patients, 84 patients *CC* (wild-type) plus *CT* (heterozygous) and 10 patients *TT* homozygous, Hematopoietic toxicity in *CC/CT* and *TT* was 51/84 (60.7%) and 6/10 (60%), respectively (*p = *0.2). Platelet toxicity in *CC/CT* and *TT* was 64/84 (76.2%) and 8/10 (80%), respectively (*p = *1). Hepatotoxicity in *CC/CT* and *TT* was 34/84 (40.5%) and 6 (60%), respectively (*p = *0.3) (Table 3).

*MTHFR A1298C polymorphism and toxicity in pediatric ALL*

Regarding *MTHFR A1298C* polymorphism, there was no significant difference between the patients of different alleles suffering from anemia as MTX toxicity (*p = *0.4). MTX did not show a significant toxic effect on platelets within the patients of different alleles (*p = *0.4). The hepatotoxic effect of MTX did not show any significant difference between the patients with different alleles (*p = *0.7) (Table 2).

The *MTHFR A1298C genotype* was done among the 94 patients where 59 patients were *AA* (*wild-type*) and 35 patients were *CC *(*homozygous*) plus *AC* (*heterozygous*). Hematopoietic toxicity in *CC/AC* and *AA* was 24/35 (68.6%) and 33/59 (55.9%), respectively (*p = *0.2). Platelet toxicity in *CC/AC* and *AA* was 29/35 (82.9%) and 43/59 (72.9%), respectively (*p = *0.3). Hepatotoxicity in *CC/AC* and *AA* was 13/35 (37.1%) and 27/59 (45.8%), respectively (*p = *0.5) (Table 3).

## Discussion

Allele frequencies of the present study are slightly different from the study done by Zidan *et al.* on the Egyptian population who showed the *c.677C>T CC*, *CT* and *TT *alleles frequencies were 26.3%, 37.5%, and 36.2%, respectively, and *c.1298A>C AA, AC*, and *CC* alleles frequencies were 16.25%, 40%, and 43.75%, respectively (19). These differences may be due to the low number of patients included in both studies and also obvious heterogeneity of the Egyptian population, and large-scale genetic studies may clarify the exact gene distribution. 

There are no reliable tests or assessments that can predict MTX toxicity, a better understanding of its pharmacology can be done by using the pharmacogenetics tools to study the effect of genetic differences in the action of enzymes involved in MTX metabolic pathways that may play a role in determining its relapse and toxicity (20). 

In the present study, no significant associations were detected between *MTHFR c.677C>T* or *c.1298A>C* polymorphisms and either overall or site-specific MTX toxicity, and either hematological or non-hematological like hepatotoxicity. These results are in accordance with studies done by Lopez-Lopez *et al.* and Ruiz-Arguelles *et al.* (5, 21). 

On the other hand, some studies showed an increased risk of developing adverse events with low dose MTX based therapy among rheumatoid arthritis patients (22, 23). 

In Acute Leukemia patients, few studies demonstrated an association of *MTHFR C677T* and *A1298C* with mild toxicities in the form of myelosuppression, hepatic toxicity, and mucositis, as well as hepatotoxicity, whereas some studies showed its association with a decrease in toxicity rates (24- 28). Whereas de Jong showed, *MTHFR 1298AC* variant may induce MTX resistance in *ex-vivo *of lymphoblasts obtained from ALL pediatric patients (29).

There is no association between c.1298C>T gene polymorphisms and the risk of developing MTX-related toxicity in the present study. Te Loo and his colleagues agreed with our results, where they concluded that it is still unclear whether the *c.677C>T* and *c.1298A>C* genetic variants play a clinically significant role in the development of MTX-induced liver toxicity (30). Also, there was a study on rheumatoid arthritis patients (31, 32). 

*c.1298A>C* polymorphisms may decrease MTX sensitivity in lymphoblast from pediatric ALL patients (29). 

This controversy in the published results may be explained by differences in study design, sample size, the different clinical setting and schedule of treatment, pathological, clinical, and demographic pattern of the patients, the inability to control another factor, *e.g.*, folate intake or parameters to measure efficacy and toxicity (28, 31 and 33). 

The race also may play a role in these conflicting results; Martin *et al.* have reported an interaction between *C677T* and *A1298C* genetic variants and race/ethnicity on breast cancer survival (34). 

Another pilot retrospective cross-validation approach study on Caucasians and African-Americans with rheumatoid arthritis resulted in the *MTHFR* genetic variant has differential effects in these racial groups suggesting the race may significantly interact with the *C677T* variant to influence the risk of MTX toxicity (35). A similar race-specific association with MTX-related adverse events in Caucasian and African–American rheumatoid arthritis patients has also been suggested for the *A1298C* variants (36).

**Table 1:** Genotype Frequencies of the studied patients

		***677 genotype***				***1298 genotype***		
allele		*CC*	*CT*	*TT*		*AA*	*AC*	*CC*
Frequency		40 (42.6%)	44 (46.8%)	10 (10.6%)		59 (62.7%)	23 (24.5%)	12 (12.8%)

**Table 2:** Clinical Risk factors and events

		***677 genotype***					***1298 genotype***			
		*CC*	*CT*	*TT*	*P*		*AA*	*AC*	*CC*	*P*
Haemoglobin (Normal)		16 (43.2%)	17 (45.9%)	4 (10.8%)	0.99		26 (70.3%)	8 (21.6%)	3 (8.1%)	0.4
Haemoglobin (Anaemia)		24 (42.1%)	27 (47.4%)	6 (10.5%)			33 (57.9%)	15 (26.3%)	9 (15.8%)	
Normal Platelets		7 (31.8%)	13 (59.1%)	2 (9.1%)	0.4		16 (72.8%)	3 (13.6%)	3 (13.6%)	0.4
Thrombocytopenia		33 (45.8%)	31 (43.1%)	8 (11.1%)			43 (59.7%)	20 (27.8%)	9 (12.5%)	
No Hepatic toxicity		26 (48.1%)	22 (40.7%)	6 (11.1%)	0.4		32 (59.2%)	15 (27.8%)	7 (13%)	0.7
Hepatic toxicity		14 (35%)	22 (55%)	4 (10%)			27 (67.5%)	8 (20%)	5 (12.5%)	

**Table 3:** MTX toxicity among wild plus heterozygous versus homozygous all patients

	**Hematopoietic toxicity**	***P*** **-value**	**Platelets toxicity**	***P*** **-value**	**Hepatotoxicity**	***P*** **-value**
***MTHFR 677 *** **genotype**						
*CC* (wild-type) plus *CT* (heterozygous) (n = 84)	51 (60.7%)	0.2	64 (76.2%)	1	34 (40.5%)	0.3
*TT* (homozygous) (n = 10)	6 (60%)		8 (80%)		6 (60%)	
***MTHFR*** ***1298 *genotype**						
*CC* (homozygous) plus *AC* (heterozygous) (n = 35)	24 (68.6%)	0.2	29 (82.9%)	0.3	13 (37.1%)	0.5
*AA* (wild-type) (n = 59)	33 (55.9%)		43 (72.9%)		27 (45.8%)	

## Conclusion

From the present results, we can conclude that neither *c677C>T* nor *c128A>C* polymorphisms had a role in hematopoietic or hepatic toxicity. Another wide-scale study may be needed to confirm these findings among the Egyptian population.
